# Tocilizumab unfolds colo-protective and immunomodulatory effect in experimentally induced ulcerative colitis via mitigating autophagy and ER stress signaling

**DOI:** 10.1007/s10787-024-01527-7

**Published:** 2024-08-12

**Authors:** Omnia A. Younes, Doaa M. Elsherbiny, Diana M. F. Hanna, Amany M. Gad, Samar S. Azab

**Affiliations:** 1 Biologicals Unit at General Administration of Clinical Studies, Egyptian Drug Authority (EDA), Giza, Egypt; 2 Pharmacology and Toxicology Department, Faculty of Pharmacy, Ain Shams University, Cairo, Egypt; 3 Department of Pharmacology, Egyptian Drug Authority (EDA), Formerly NODCAR, Giza, Egypt; 4 Department of Pharmacology and Toxicology, Faculty of Pharmacy, Sinai University Kantara Branch, Ismailia, Egypt

**Keywords:** Ulcerative colitis, Tocilizumab, Inflammatory bowel disease, Ustekinumab, Autophagy, Immunomodulatory

## Abstract

Ulcerative colitis (UC) is an idiopathic, chronic, relapsing inflammatory bowel disease (IBD), characterized by chronic inflammation of the gastrointestinal tract. The pathophysiology of UC is complicated and involves several factors including immune, genetic, and environmental factors. Recently, a huge amount of research has concentrated on the role of interleukins including interleukin-6 (IL-6) in its pathophysiology. Thus, this study aims to examine the colo-protective and immunomodulatory effect of Tocilizumab (TCZ) in an experimental model of dextran sulfate sodium (DSS) induced UC. In the current study, we analyzed the inflammatory, immunomodulatory, apoptotic, autophagy, and endoplasmic reticulum (ER) stress markers and other clinical features including stool consistency, rectal bleeding, and edema markers in rats. Our results showed that induction of colitis caused bloody diarrhea and increased IL-6 levels. Treatment with TCZ significantly ameliorated DSS-induced injury via decreasing inflammatory markers of colon injury (IL-6), signal transducer and activator of transcription-3 (STAT-3), and C-reactive protein (CRP). Furthermore, TCZ attenuated the apoptotic marker (caspase-3), and down-regulated endoplasmic reticulum stress sensor proteins (inositol- requiring transmembrane kinase endonuclease-1 (IRE-1) and activated transcription factor-6 (ATF-6)) and autophagy proteins (autophagy-related 16-like protein 1 (ATG16L1) and nucleotide-binding oligomerization domain-containing protein-2 (NOD2)), as compared to DSS group. Altogether, the current data suggest TCZ to be a promising protective therapy against UC.

**Graphical Abstract:**

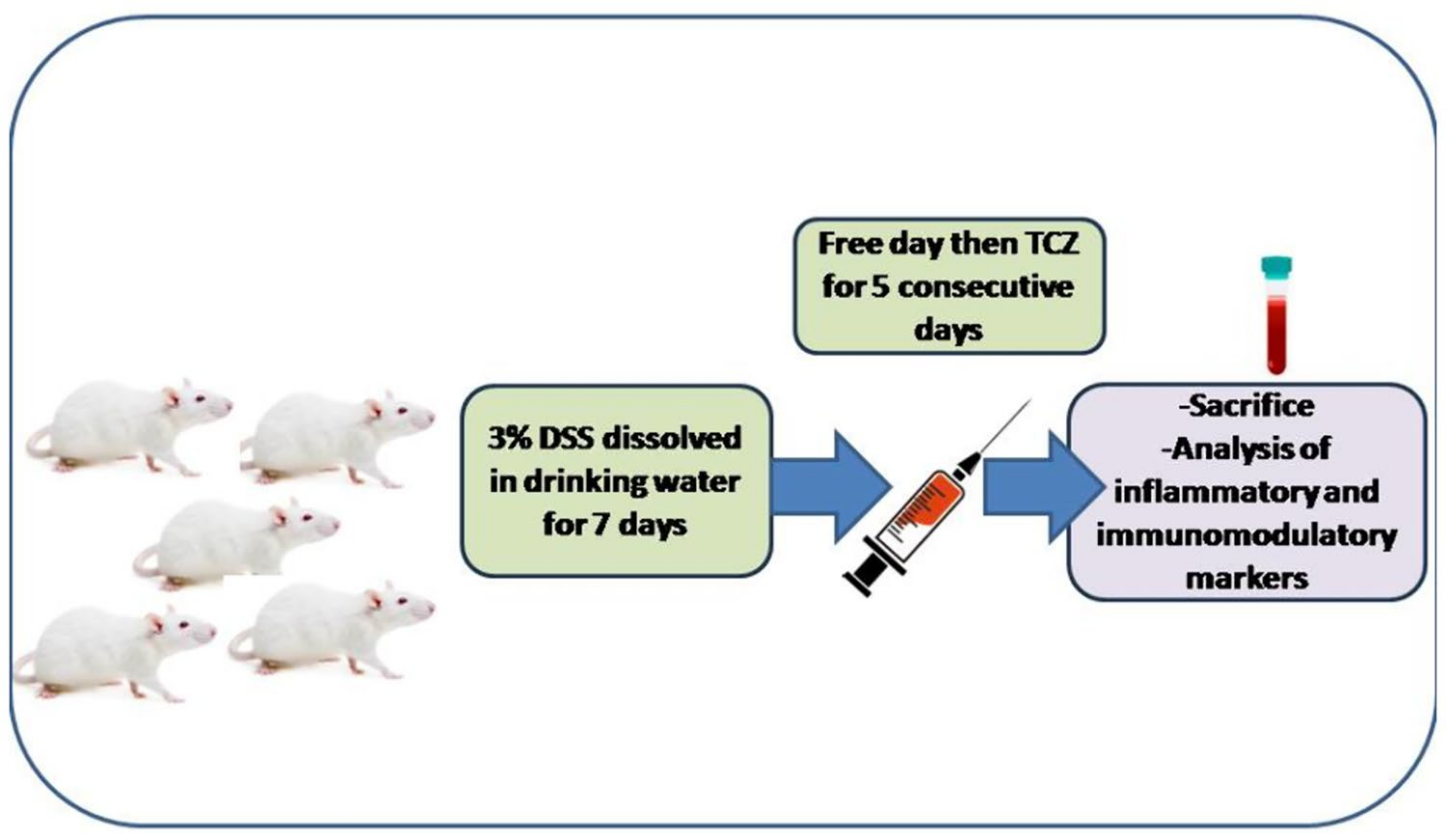

## Introduction

Inflammatory bowel diseases (IBDs) are idiopathic, chronic, inflammatory autoimmune disorders characterized by relapsing–remitting inflammation of the gastrointestinal tract. They encompass two inflammatory diseases: Crohn’s disease (CD) and ulcerative colitis (UC) (Sartor 2006). UC is a disorder of unknown cause that affects the colonic mucosa and is clinically characterized by diarrhea, bloody stools, frequent bowel movements, abdominal pain, fever, malnutrition, and weight loss (Podolsky 2002). The severity of the disease may also be quite histologically variable ranging from minimal to severe ulceration and dysplasia ending in the potential development of carcinoma (Wang and Fang 2014).

The pathophysiology of UC is complicated and involves several factors (Peterson and Artis 2014) including immune, genetic, and environmental factors (Kaistha and Levine 2014; Guan 2019). The intestinal epithelial barrier normally protects the body against pathogens as it is the first immune defense to elicit immune responses against luminal antigens (Matricon et al. 2010). This is regulated by pattern recognition receptors, such as Toll-like receptors (TLRs) expressed normally by intestinal epithelial cells and underlying immune cells. TLR4 activation in response to injury or infection results in the induction of pro-inflammatory cytokines such as tumor necrosis factor(TNF-α), interleukin-6 (IL-6), and IL-1β (Ahluwalia et al. 2018) which leads to increased local endothelial expression of intracellular adhesion molecule-1 (ICAM-1),and vascular cell adhesion molecule-1 (VCAM-1) necessary for circulating cells to be able to stick to activated endothelium. TNF-α also increases immune response, Paneth cell necrosis, and stimulates intestinal epithelial cell death (Rijcken et al. 2002).

Medical therapies, as well as surgical intervention, are the current modalities for the treatment of UC (Antunes et al. 2021). These therapies involve anti-inflammatory corticosteroids which are commonly used, and disease-modifying anti-rheumatic drugs (DMARDs) such as sulfasalazine (Das and Farag 2000; Seyedian et al. 2019). However, they suffer from short-term clinical improvement, poor patient compliance as well as several side effects including headache, nausea, and hypersensitivity causing fever and rash (Kayal and Shah 2019). Other available treatments are immune suppressors (such as azathioprine, mercaptopurine, tacrolimus, and cyclosporine) which have limited efficacy, and multiple cardiovascular side effects including atrial fibrillation and prolonged QT interval, and other rare effects such as angina, hypotension, venous thrombosis, and cardiogenic shock (Garud and Peppercorn 2009).

Hence, there is a current and urgent need to explore newer drugs to manage UC in a more efficient and less toxic way. This conclusion led to the start of using biological therapies (Valatas et al. 2013; Allocca et al. 2018). Anti-TNF-α monoclonal antibodies (such as golimumab, adalimumab, and infliximab) represent an efficacious UC treatment option (Pedersen et al. 2014; Martina and Ana 2019). Nevertheless, a considerable proportion of patients lose response due to the formation of anti-drug antibodies (ADA) (Strand et al. 2017). In addition, they have many cardiovascular side effects as they can lead to an increased risk of death and worsening heart failure (HF) in moderate to severe HF patients (Sinh and Cross 2021). Golimumab was reported to cause congestive heart failure (Padda, et al. 2022). Adalimumab has also been shown to induce tachyarrhythmia, palpitation, and cardiomyopathy (Toufaily A. 2020). Likewise, infliximab can cause atrioventricular block, bradycardia, and heart failure (Sote et al. 2007). Subsequently, other drugs were used, such as Ustekinumab (UST), a monoclonal antibody directed against the P40 subunit of IL-12 and IL-23 (Benson et al. 2011; Chaparro et al. 2021), however, it was associated with unexpected increases in cardiovascular problems (e.g., congestive heart failure) (Beroukhim et al. 2015; Morgenweck et al. 2022).

Importantly, Interleukin-6 (IL-6) is one of the pro-Inflammatory cytokines involved in the inflammation process of UC (Wang and Sun 2014) and one of the molecules involved in many cardiovascular disorders. Consequently, a rationale strategy for managing UC could be realized by blocking signal transduction of IL-6 as it is involved in the pathogenesis of UC through regulating immune response, modulating the intestinal barrier, and interacting with the intestinal microbiota (Tie et al. 2023). The anti-IL-6 antibody tocilizumab (TCZ) is currently used for rheumatoid arthritis treatment (Mihara et al. 2011), systemic juvenile idiopathic arthritis (Yokota et al. 2012) and polyarticular juvenile idiopathic polyarthritis (Turnier and Brunner 2016). TCZ has shown positive effects on UC in a patient with coexisting ulcerative colitis and rheumatoid arthritis (Szeto et al. 2016). Interestingly, TCZ has demonstrated a cardiovascular safety profile compared to other biologics (Castagné et al. 2019).

Therefore, the current study aims to first screen for the colo-protective and immunomodulatory dose of TCZ in an experimentally induced UC. Second, to investigate the underlying molecular mechanisms mediating this protective effect. Third, to evaluate the cardiac effect of TCZ in the tested experimental model.

## Materials and methods

### Chemicals

Dextran sulfate sodium (DSS, molecular weight of approximately 40,000 Daltons) was purchased from TdB consultancy (Uppsala, Sweden). It is a polyanionic derivative of dextran with a chemical formula of (C6H7Na3O14S3). Tocilizumab (TCZ) vials were the product of Hoffmann-La Roche Co. (Actemra®) and Ustekinumab (UST) was the product of Janssen Co. (Stelara®). All other chemicals were of the highest commercially available grade.

### Animals

Male albino Wistar rats weighing approximately 200–250 g were obtained from the animal house facility of the National Organization for Drug Control and Research (NODCAR), Cairo, Egypt. Animals were allowed to acclimatize for 1 week before starting the experiment. Then, were housed in separate polystyrene cages under a constant temperature of 25 °C and a 12-h light/dark cycle and were fed with standard show pellets ad libitum.

### Ethical statement

Animal handling and experimental procedures were performed per the guidelines of “International Ethical Guidelines” concerning the care and use of laboratory animals, and the experimental protocol was approved by the scientific research ethics committee of the Faculty of Pharmacy, Ain Shams University (ENREC, ASU2019-263).

### Experimental design

The experiment lasted for 2 weeks. Fifty-four Wistar rats were randomly distributed in 9 groups to have six rats per group which are outlined below:

*Group I: *Control received distilled water, for the entire period of the study.

*Group II:* Induction with DSS; rats received 3% DSS dissolved in drinking water for the first 7 days, followed by 2 booster doses every third day of administration, i.e., on day 10 and day 13 alternating with drinking water for the total period of the study 14 days, as previously described in Morsy et al. (2019).

*Groups III, IV, and V: *In treatment groups, rats received 3% DSS dissolved in drinking water for the first 7 days followed by 1 day free then received TCZ intraperitoneal (i.p.) in three different doses (5 mg/kg, 10 mg/kg, and 20 mg/kg) for 5 consecutive days from day 9 to day 13. The treatment schedule was based on previous studies (Taniguchi et al. 1998; Triantafillidis, et al. 2005) and the treatment doses were selected based on previous studies (Abdel-Maged et al. 2018; Zhu et al. 2023).

*Groups VI and VII:* Drug-alone groups, both groups received only treatment drug TCZ i.p. in two different doses (10 mg/kg and 20 mg/kg) for 5 consecutive days starting from day 9 to day 13.

*Groups VIII and IX:* Standard comparator groups, rats received 3% DSS dissolved in drinking water for the first 7 days followed by 1 day free then received UST i.p. in two different doses (10 mg/kg and 20 mg/kg) for 5 consecutive days starting from day 9 to day 13.

During the screening phase, the clinical features including stool consistency, and rectal bleeding were observed. Scoring was performed to evaluate stages of disease activity and severity according to the disease activity index (DAI) scoring system (Pabla and Schwartz 2020): stool consistency (0 = normal, 1 = loose, 2 = watery diarrhea, 3 = slimy diarrhea with little blood, 4 = severe watery diarrhea with blood), and presence of blood in the stool (0 = no blood, 2 = presence of blood by ColoScreen, 4 = visible blood).

**Fig. 1:**
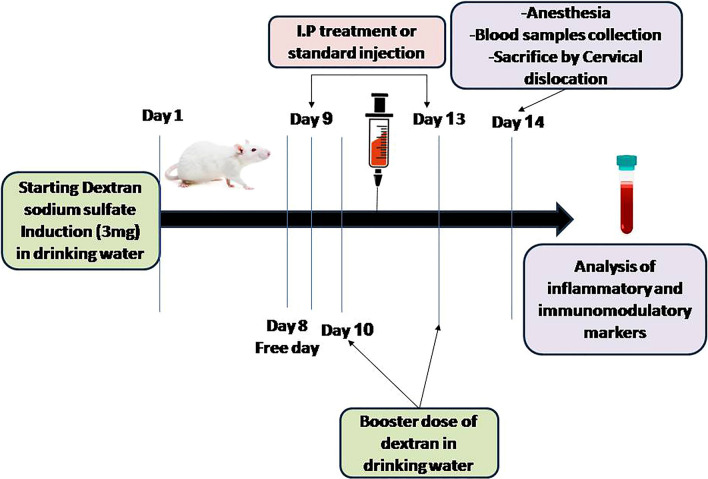
Graphical depiction of experimental design and timeline: 3% Dextran sodium sulfate (Dss) was dissolved in distilled drinking water from day 1 to day 7; then, two booster doses were administered on days 10 and 13 of the experiment. Tocilizumab (TCZ) or Ustekinumab (UST) were injected i.p. in three different doses for 5 consecutive days from day 9 to 13. On day 14, blood samples were withdrawn from the retro-orbital plexus after anesthesia and then rats were killed by cervical dislocation

Blood samples were collected from the retro-orbital plexus and serum samples were then separated by centrifugation at 1000g for 10 min and stored at – 80 °C for assessment of cardiac markers. Then, rats were anesthetized andsacrificed by cervical dislocation. Colon weight and length were measured as edema markers in addition to macroscopic and microscopic examination. Colon and heart tissue samples were excised from all groups and served for histological examination of ulcers and inflammatory cell infiltration. Then, other colon tissue samples were homogenized, and the homogenates were used for the assessment of inflammatory, immunomodulatory, apoptotic, autophagy, and ER stress markers in the mechanistic phase. Experimental design and timelines are illustrated in Fig. 1.

### Histopathological investigation and scorings

Colon tissue and heart tissue samples were fixed in 10% formalin and partitioned into paraffin slices of 4 µm thickness. Slices were then stained with hematoxylin and eosin (H & E) for both colon and heart tissues and Alcian blue for colon specimens only. That was followed by histopathological investigation under the light microscope Olympus BX-50 (Olympus Corporation, Japan). Histopathology images were taken at a magnification of × 16 in the case of Hematoxylin and eosin (H&E) stain and × 40 in the case of Alcian blue stain by a digital camera attached to the microscope. The severity of histopathological alterations was scored, and the following hallmarks were investigated in the colon tissue: mucosal ulceration, hyperemia, and inflammatory cell infiltration in the lamina propria and mucosa. Besides, heart tissues were investigated for any histopathological changes such as alterations or degenerations in the myocardium bundles, blood vessels, and myocardial muscle cells.

### Assessment of inflammatory and immunomodulatory markers

Enzyme-linked immunosorbent assay (ELISA) technique was carried out for estimation of the following markers in the colon: signal transducer and activator of transcription-3 (STAT-3, quantitative sandwich ELISA, MBS2515874, Mybiosource, USA), IL-6 (quantitative sandwich ELISA, R6000B, Quantikine R and D, USA and Canada), in addition to cardiac C-reactive protein (CRP, Sandwich ELISA, MBS2508830, Mybiosource, USA). Results were expressed as pg/mg protein for IL-6, ng/mg protein for STAT-3, and ng/ml for CRP.

### Assessment of apoptotic and cardiac markers

Colorimetric assays were performed for estimation of caspase-3 (E-CK-A311, Elabscience, USA) in the colon, in addition to cardiac lactate dehydrogenase (LDH; K726-500, BioVision, USA) and cardiac creatine kinase-myocardial band (CK-MB; CKM108050, BioMed, Germany). Results were expressed as U/ml for LDH and CK-MB.

### Assessment of ER stress and autophagy markers

Estimation of inositol-requiring transmembrane kinase endonuclease-1 (IRE-1), activated transcription factor-6 (ATF-6), nucleotide-binding oligomerization domain-containing protein-2 (NOD2), and autophagy-related 16-like protein 1 (ATG16L1), in the colon, was carried out using Western blotting technique. Proteins were separated according to their molecular weight by sodium dodecyl sulfate–polyacrylamide gel electrophoresis (SDS-PAGE) and then transferred to nitrocellulose membranes. That was followed by incubation with primary antibodies (1:2000, CAT: PA5-22,252, Thermo Fisher, USA), and secondary horseradish peroxidase (HRP)-conjugated antibodies (CAT: ab6721, Novus Biologicals, USA). The separation was visualized using stain-free technology and image analysis to read the band intensity using a ChemiDoc TM imager.

### Statistical analysis

Statistical analyses were performed using Graph Pad Instat Software, Inc. La Jolla, CA, USA, version 3.00 and graphs were plotted using Graph Pad Prism Software, version 8.00 Inc. La Jolla, CA, USA. The measured parameters were expressed as mean ± Standard of deviation (SD). Stool consistency, bloody diarrhea, and macroscopic scoring were analyzed using the Kruskal–Wallis test followed by Dunn's post hoc test. The rest of the data were analyzed using a one-way analysis of variance (ANOVA) followed by Tukey's post hoc test. The minimal level of significance was identified at *P* < 0.05. Figures were labeled with a, b: statistically significant from Control and Dss groups, respectively.

## Results

### Colo-protective effects

#### Symptomatic markers

Dss significantly reduced colon length compared to the control group, while TCZ (10 mg/kg, 20 mg/kg) and UST (10 mg/kg) significantly increased colon length compared to the Dss-induced group restoring them to normal.

TCZ-treated group (20 mg/kg) and TCZ-alone (10 mg/kg, 20 mg/kg) reduced lesion score significantly compared to Dss-induced group, while UST did not induce any significant change. These symptomatic markers are presented in Table 1.

**Table 1:** Effect of different experimental groups on colon lesion parameters in male Wister rats with Dss-induced ulcerative colitis

Parameters	Experimental groups								
	Control	Dss	TCZ + Dss			TCZ only		UST + Dss	
			TCZ 5 mg	TCZ 10 mg	TCZ 20 mg	10 mg	20 mg	10 mg	20 mg
Colon weight	3.308 ± 5.3	1.4 ± 0.17	1.35 ± 0.2	1.585 ± 0.25	1.591 ± 0.3	1.66 ± 0.3	1.65 ± 0.24	2.048 ± 0.2	1.675 ± 0.2
Colon length	17.92 ± 2.2	13.94 ± 1.8^a^	16.63 ± 1.09	17.5 ± 1.63^b^	17.06 ± 1.5	14.63 ± 2.8	18.5 ± 1^b^	18 ± 1.8^b^	16.25 ± 2.9
Colon weight/length	0.08125 ± 0.01	0.0995 ± 0.02	0.0759 ± 0.01	0.08975 ± 0.02	0.09238 ± 0.02	0.117 ± 0.04^a,c^	0.087 ± 0.01	0.1075 ± 0.01	0.104 ± 0.02
Lesion score	0	3.375 ± 1.1^a^	1 ± 1.06	1.25 ± 1.06	0.25 ± 0.7^**b**^	0^b^	0^b^	1 ± 1.2	1.5 ± 1

^**a**^Statistical significance as compared to the control

^**b**^Statistical significance as compared to the dextran sodium sulfate (Dss)-treated group

^**c**^Statistical significance as compared to the Tocilizumab (TCZ)-treated group

Dss significantly increased bloody diarrhea compared to the control group, while TCZ (10 mg/kg, 20 mg/kg) significantly reduced it compared to the Dss group restoring it to normal. The effect of different experimental groups on disease activity index (DAI) scores is presented in Table 2.

**Table 2:** Effect of different experimental groups on disease activity index (DAI) scores in male Wister rats with Dss-induced ulcerative colitis

	Experimental groups									
	Control	Dss	TCZ + Dss			TCZ only			UST + Dss	
Parameters			TCZ 5 mg	TCZ 10 mg	TCZ 20 mg	10 mg	20 mg	10 mg		20 mg
Stool consistency	1 ± 0	1.625 ± 0.9	0.875 ± 0.83	0.375 ± 0.744	0^**a**^^**,b**^	0^**b**^	0^**b**^	0^**b**^		0.75 ± 0.5
Bloody diarrhea	0 ± 0	2 ± 1.69^**a**^	0.5 ± 0.53	0.375 ± 0.5	0 ± 0^b^	0 ± 0	0 ± 0	0.25 ± 0.5		0.5 ± 0.57

^**a**^Statistical significance as compared to the control

^**b**^Statistical significance as compared to the dextran sodium sulfate (Dss)-treated group

#### Macroscopic examination/scoring

**Fig. 2:**
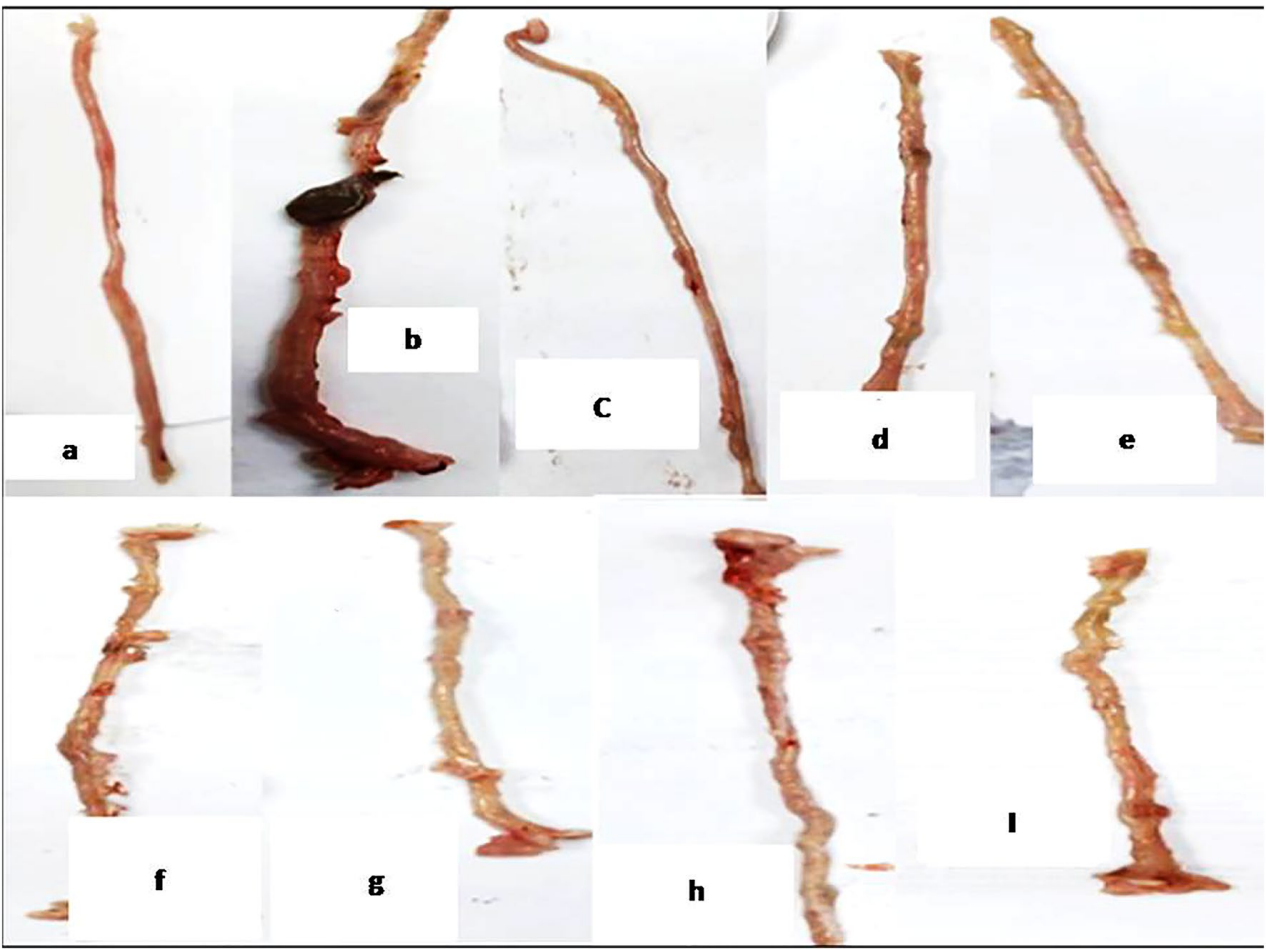
Macroscopic examination of colonic samples from different experimental groups: **a** rats in 1st group served as the control group showing normal colon tissue. **b** Rats in the Dextran sodium sulfate (Dss) induction group showed ulceration, congestion, and edema. **c** Low dose Tocilizumab-treated group showing light ulceration of colon tissue. **d, e** Medium and high-dose Tocilizumab-treated groups showed no ulcers. **f, g** Medium and high doses of Tocilizumab alone showed no ulcers. **h, l** Ustekinumab-treated groups showed no ulceration of colon tissue with both medium and high-dose groups

As compared to the normal appearance of colon tissues from control rats, ulceration, congestion, and edema were observed in those of the diseased group. Colon tissues from TCZ-treated rats demonstrated light ulceration with low-dose (5 mg/kg) treatment, while no ulcers could be observed with medium (10 mg/kg) and high (20 mg/kg)-dose treatments. Colons from UST (10 mg/kg and 20 mg/kg)-treated groups displayed similar findings to those from TCZ (10 mg/kg and 20 mg/kg)-treated rats, respectively. All these observations are demonstrated in Fig. 2.

#### Microscopic histopathological examination

**Fig. 3:**
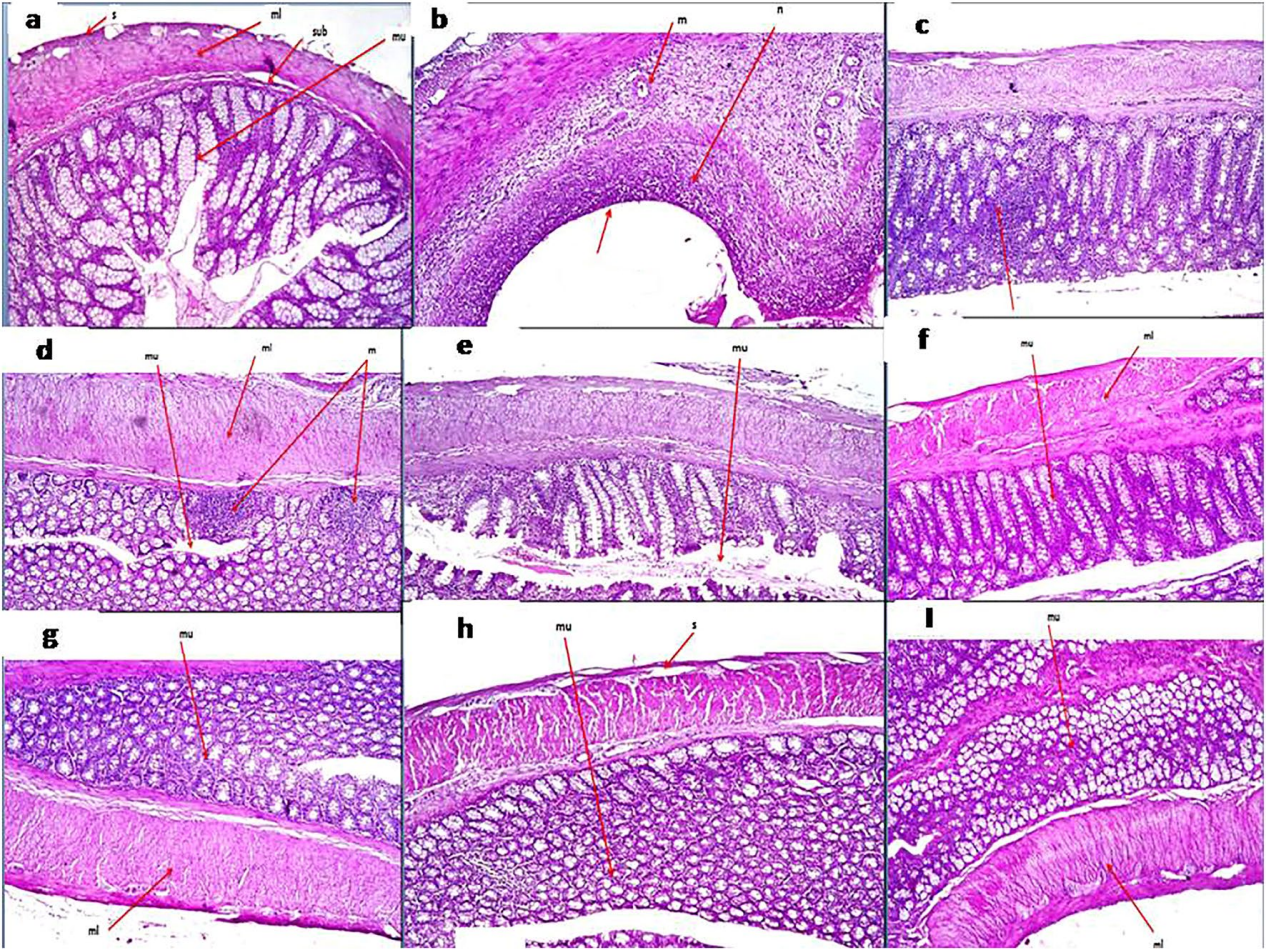
Photomicrographs of colonic sections of different experimental groups stained by H&E (All images were taken at a magnification of × 16 by a digital camera attached to the microscope): **a** control group showing normal histopathological structure. **b**. Dextran sodium sulfate (Dss) induction group showing focal necrosis, ulceration with inflammatory cells infiltration, thickening, and edema. **c** Tocilizumab (TCZ)-treated groups showing focal inflammatory cell infiltration with 5 mg/kg dose, **d** focal inflammatory aggregation with a 10 mg/kg dose. **e** Very few inflammatory cell aggregations with 20 mg/kg dose. **f, g** TCZ-alone group showed no histopathological alteration with both 10 mg/kg and 20 mg doses. **h, I** Ustekinumab-treated groups showed focal few inflammatory cell infiltration with a 10 mg dose and no histopathological alteration with a 20 mg/kg dose

**Fig. 4:**
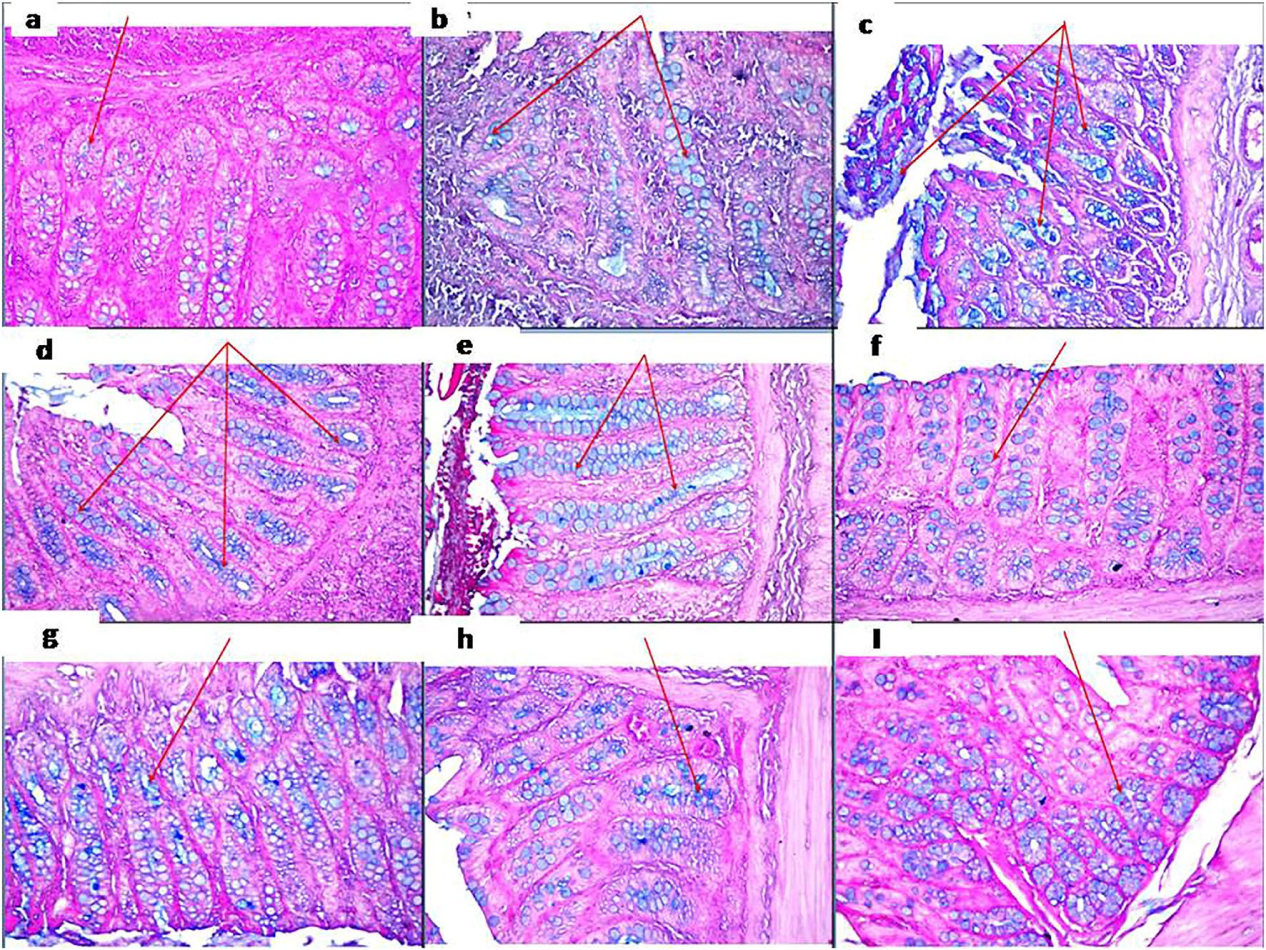
Photomicrographs of colonic sections of different experimental groups stained by Alcian blue (all images were taken at a magnification of × 40 by a digital camera attached to the microscope): **a** the control group showed moderate goblet cell formation. **b** Dextran sodium sulfate (Dss) showed marked mucin depletion and mild goblet cell formation. **c, d, e** Tocilizumab (TCZ)-treated groups showing strong goblet cell formation. **f, g** TCZ-alone group showing moderate goblet cell formation. **h, I** Ustekinumab-treated groups showed moderate goblet cell formation

Colon tissue specimens from rats stained by H&E demonstrated normal histological structure of the mucosal layer with lamina propria, submucosa, muscularis, and serosa in the control group. In the disease group, focal necrosis and ulceration with inflammatory cell infiltration were detected in the mucosa and submucosal layers. The muscularis showed hypertrophy with few inflammatory cells’ infiltration, thickening, and edema were observed in the serosal layer. TCZ-treated rats demonstrated focal inflammatory cell infiltration in the lamina propria of the mucosal layer with low dose (5 mg/kg), focal inflammatory cells aggregation in the lamina propria of the mucosa with medium dose (10 mg/kg), and very few inflammatory cells infiltration in the lamina propria of the mucosa with high dose (20 mg/kg). No histopathological alterations were detected in TCZ-alone groups. UST-treated rats showed focal inflammatory cell infiltration in lamina propria of the mucosal layer with a medium dose (10 mg/kg), while no histopathological alterations were observed with the high-dose (20 mg/kg) group. Histopathological examinations of colon specimens stained by H& E from all experimental groups are demonstrated in Fig. 3. Alcian blue-stained colon tissue specimens demonstrated different severity of goblet cell formation in a mucosal layer in colon tissue of different experimental groups as shown in Table 3 and Fig. 4 and the scoring of histopathological alteration is presented in Table 4.

**Table 3:** Representative of severity of goblet cell formation in mucosal layer in colon tissue in all experimental groups by Alcian blue stain

Parameters	Experimental groups								
	Control	Dss	TCZ + Dss			TCZ Only		UST + Dss	
			TCZ mg	TCZ 10 mg	TCZ 20 mg	10 mg	20 mg	10 mg	20 mg
Appearance of blue color replacing the mucous in the mucosal epithelium and glands showing:	++	+	+++	+++	+++	++	++	++	++

+++: Severe

++: Moderate

+: Mild

**Table 4:** Severity of histopathological alterations in colon and heart of Wistar rats in experimental groups

	Histopathological alteration(s)	Group No								
		1	2	3	4	5	6	7	8	9
Colon	Ulcerative colitis	−	+ + +	−		−	−	−	−	−
	Light colitis	−	+	+	+	−	−	−	−	−
Heart	Congestion	−	+	−	+ +	+	−	−	−	−
	Myocardial degeneration	−	+	+	+ +	−	−	−	−	−

+++ Severe

++ Moderate

+ Mild

Nil

Based on histopathological results in the screening phase, it was decided to test only the high-dose group of both treatment (TCZ) and standard (UST) in the mechanistic phase.

### Inflammatory markers

**Fig. 5:**
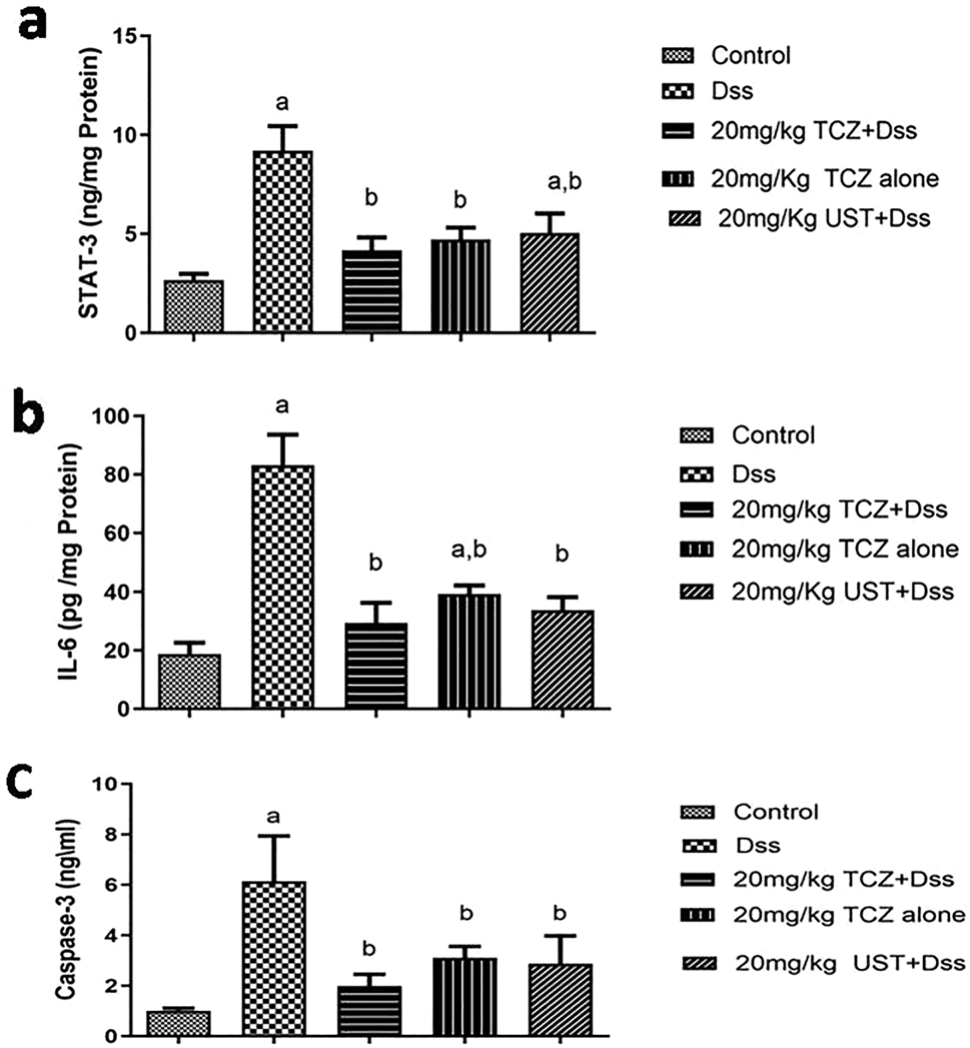
Inflammatory, immunomodulatory, and apoptotic markers levels in colonic sections of different treatment groups: **a** the control group showed negative expression of the signal transducer and activator of transcription-3 (STAT-3). The dextran sodium sulfate (Dss) group shows significantly higher expression levels in colon specimens. While high doses of Tocilizumab (TCZ) or Ustekinumab (UST)-treated groups showed minimal expression levels. Data represented with *P* < 0.0001 and *F* = 25.77 (one-way ANOVA followed by Tukey’s multiple comparison post hoc test). **b** The control group showed negative expression of interleukin-6 (IL-6). Dss group showed massive expression in colon specimens, while TCZ-treated groups, TCZ-alone, and UST-treated groups showed minimal expression. The high dose of the group showed the highest reduction levels. Data represented with *P* < 0.0001 and *F* = 46.56 (one-way ANOVA followed by Tukey’s multiple comparison post hoc test). **c** Control group showing negative expression of Caspase-3. The Dss group showed high expression in colon specimens while high dose of TCZ-treated groups showed minimal expression levels. Data represented with *P* < 0.001 and *F* = 11.34 (one-way ANOVA followed by Tukey’s multiple comparison post hoc test)

As presented in Fig. 5A, the colon tissues of rats in the DSS-treated group showed significantly higher expression levels of STAT-3 as compared to control rats by 344%. In contrast, high doses (20 mg/kg) of TCZ-treated, and UST-treated groups significantly reduced the expression levels of STAT-3 by 45.3% and 55%, respectively, as compared to the diseased group. No significant differences were shown between TCZ-treated and UST-treated groups.

### Immunomodulatory markers

Analysis of IL-6 levels revealed massive expression levels (by 443.5%) in diseased colons as compared to control samples (Fig. 5B). As compared to the disease-induced group, IL-6 expression levels were decreased by 35.3% and 40.5% in TCZ-treated and UST-treated groups receiving high doses of 20 mg/kg, respectively. When compared to the disease group, the group treated with TCZ (20 mg/kg) exhibited reduced expression levels of IL-6 by 56.7%, a comparable value to the control group. No significant differences could be estimated between TCZ-treated and UST-treated groups.

### Apoptosis

Evaluation of the status of apoptotic machinery in rats was achieved by measuring caspase-3. In comparison to the control group, diseased colon tissues showed significant elevation in caspase-3 levels by 606.9% as illustrated in Fig. 5C. Declined levels of caspase-3 levels by 32.6% and 46.8% were achieved in rats receiving 20 mg/kg of either TCZ treatment or UST treatment, respectively, compared to DSS-treated rats. Results show no significant differences between TCZ-treated and UST-treated groups at respective doses.

### ER stress markers

**Fig. 6:**
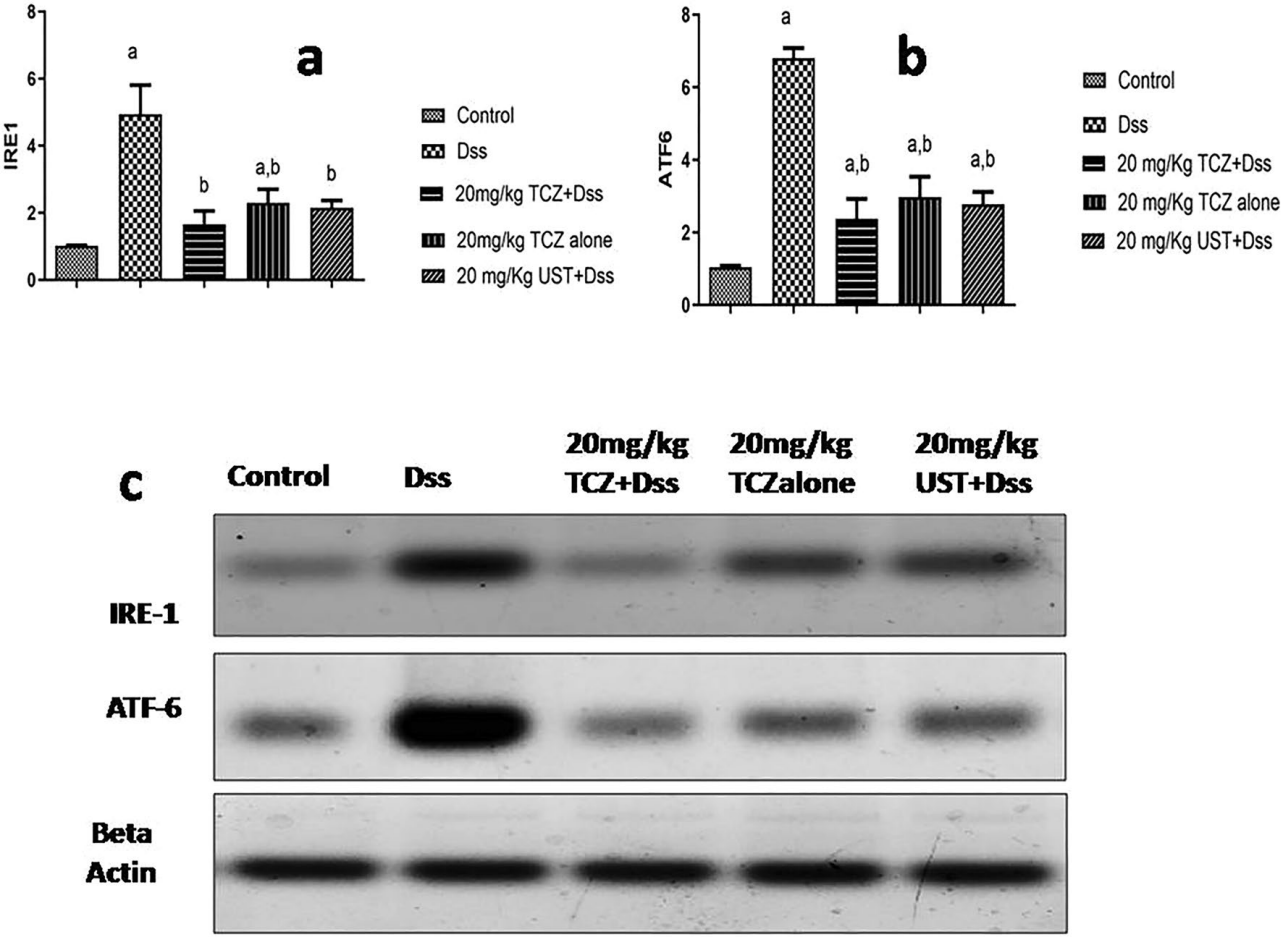
ER stress markers levels in colonic sections of different treatment groups: **a** control group showing negative expression of Inositol requiring transmembrane kinase endonuclease-1 (IRE-1). The dextran sodium sulfate (Dss) group shows massive expression in colon specimens while high doses of Tocilizumab (TCZ)-treated groups, TCZ-alone, and Ustekinumab (UST)-treated groups show reduced expression. No significant difference in expression levels in a medium dose of TCZ and UST groups. Data represented with *P* < 0.0001 and *F* (29.88) (one-way ANOVA followed by Tukey's multiple comparison post hoc test).** b** Control group showing negative expression of activated transcription factor-6 (ATF-6). Dss group shows massive expression in colon specimens, while high doses of TCZ-treated groups, TCZ-alone, and UST-treated groups show reduced expression. No significant difference in expression levels in a medium dose of TCZ and UST groups, while a significant difference level was observed in a high dose of TCZ and UST groups. Data represented with *P* < 0.0001 and *F* = 83.63 (one-way ANOVA followed by Tukey’s multiple comparison post hoc test)

Assessment of the endoplasmic reticulum stress proteins, namely IRE-1 and ATF-6 by Western blot technique (Fig. 6A, B) revealed upregulation of IRE-1 levels in DSS-treated rats by 485% compared to its respective control group. Significant downregulation of IRE-1 expression levels compared to the disease group was observed in TCZ (20 mg/kg) and UST (20 mg/kg) by 33.7% and 43.6%, respectively.

Results also revealed upregulation of ATF-6 marker levels in DSS-treated rats by 654.8% compared to its respective control group. Significant downregulation of ATF-6 expression levels compared to disease groups was observed in TCZ (20 mg/kg) and UST (20 mg/kg) groups by 34.7% and 40.6%, respectively.

### Autophagy markers

To evaluate the cellular mechanism “autophagy” as a consequence of ER stress, the expression levels of autophagy markers, namely ATG16L1 and NOD2 were assessed.

ATG16L1 expression levels were significantly decreased in disease-induced rats compared to its respective control group. When compared to DSS-treated rats, those treated with high doses (20 mg/kg) of TCZ and UST experienced upregulated levels of ATG16L1.

**Fig. 7:**
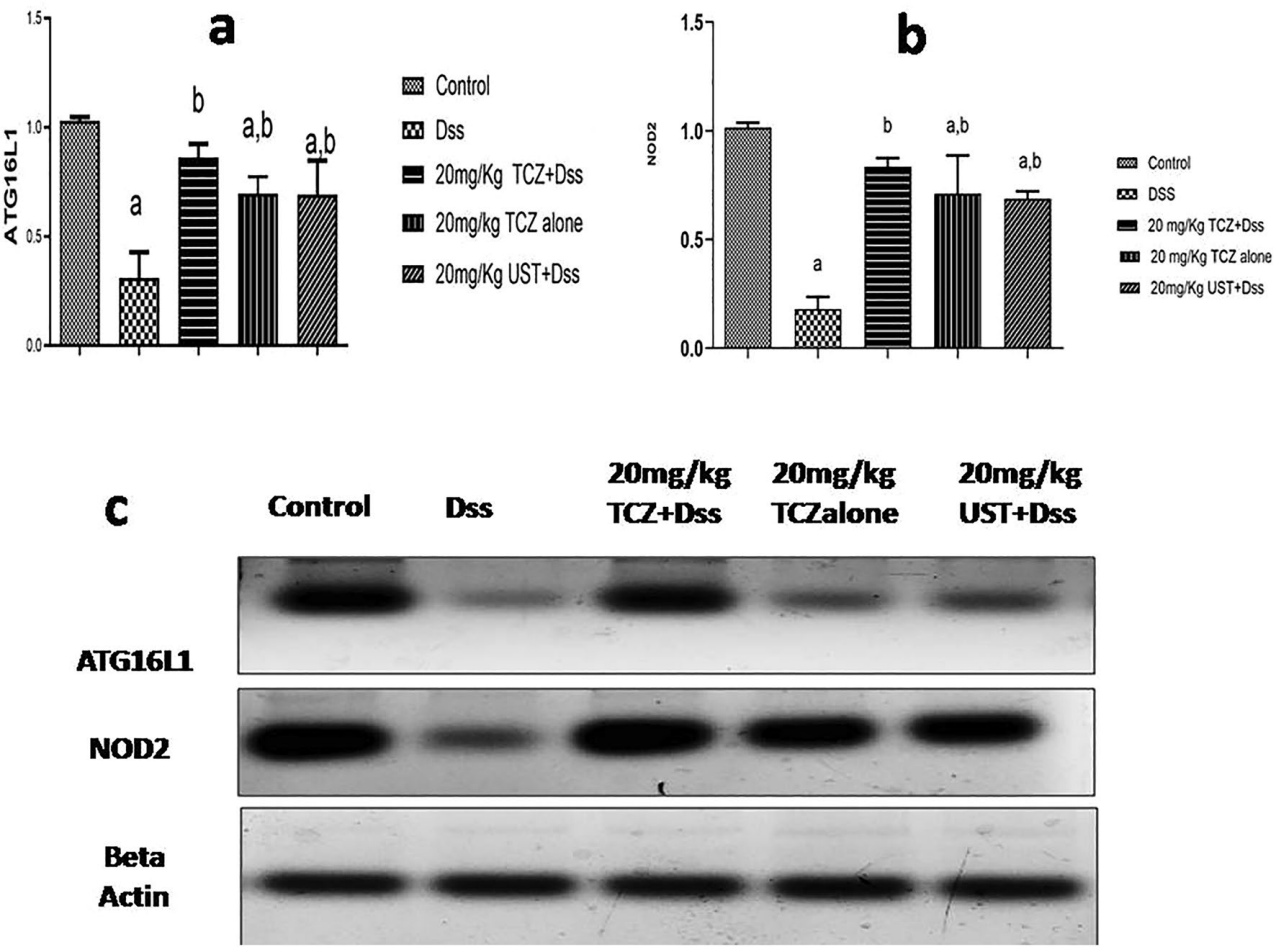
Autophagy markers levels in colonic sections of different treatment groups: **a** control group showing negative expression of autophagy-related 16-like 1 protein (ATG16L). The dextran sodium sulfate (Dss) group showed decreased levels of expression of ATG16L1 and NOD2 in colon specimens while high dose of Tocilizumab (TCZ)-treated groups, TCZ-alone and Ustekinumab (UST)-treated groups show higher expression of ATG16L1 and NOD2. Data represented with *P* < 0.0001 and *F* = 22.34 (one-way ANOVA followed by Tukey’s multiple comparison post hoc test).** b** Control group showing negative expression of nucleotide-binding oligomerization domain-containing protein-2 (NOD2). A significant difference in NOD2 levels was between the high dose of the TCZ group and the control group. Data represented with *P* < 0.0004 and *F* = 38.65 (one-way ANOVA followed by Tukey’s multiple comparison post hoc test)

The expression levels of NOD2 were significantly reduced in disease-induced rats compared to its respective control group. When compared to DSS-treated rats, those treated with high doses (20 mg/kg) of TCZ and UST experienced upregulated levels of NOD2. Results are presented in (Fig. 7A, B).

### Cardiotoxicity evaluation parameters

**Fig. 8:**
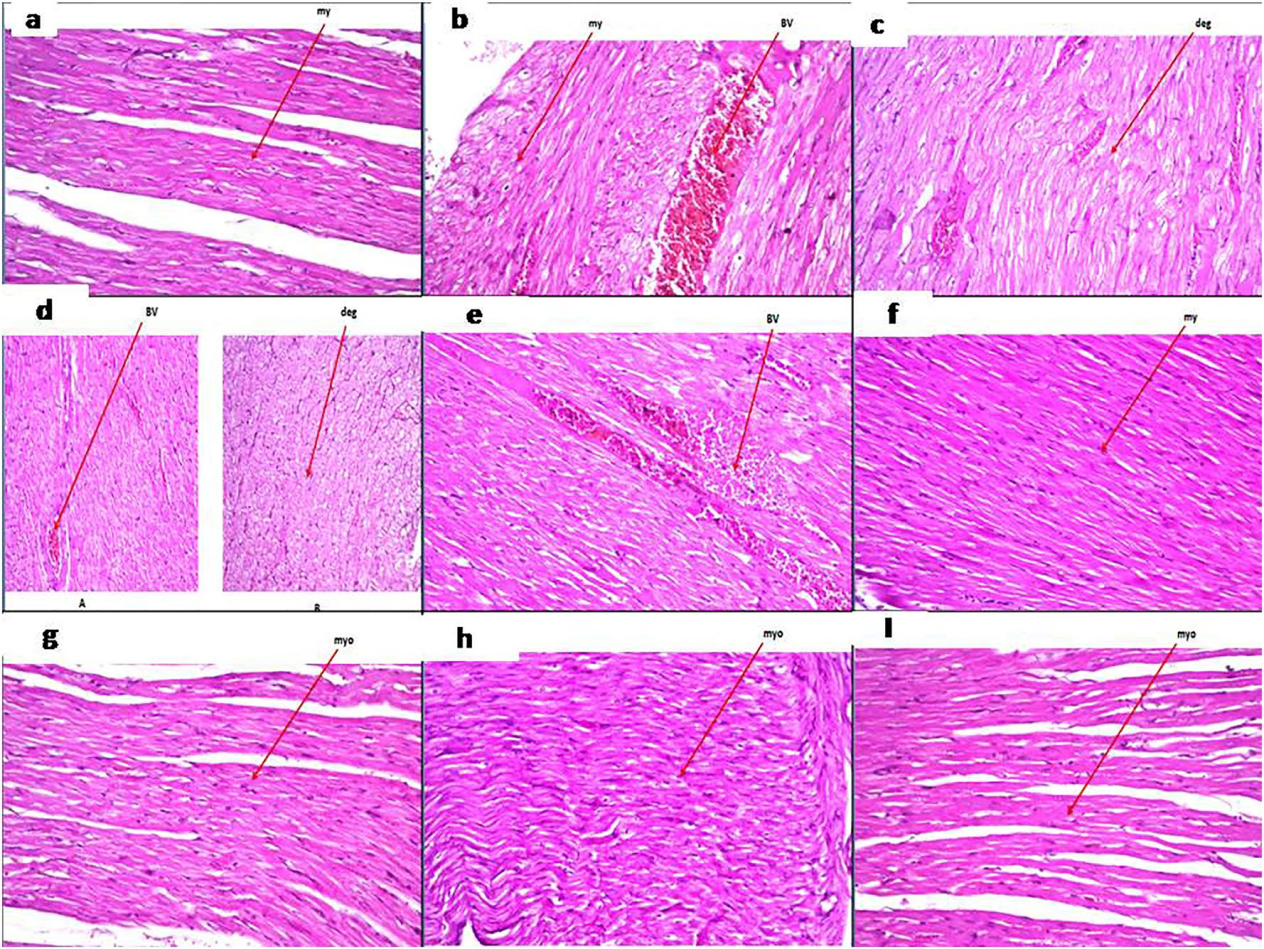
Photomicrographs of heart sections of different experimental groups stained by Hematoxylin and eosin (All images were taken at a magnification of × 40 by a digital camera attached to the microscope): **a** the control group showed normal histopathological structure of the myocardium. **b** Dextran sodium sulfate (Dss) induction group showing congestion in the myocardial blood vessels associated with degeneration in the myocardium bundles. **c** Tocilizumab** (**TCZ)-treated groups showing degenerative changes in the myocardial muscle cells with a 5 mg/kg dose. **d** Thickening of the myocardial blood vessels and sclerosis associated with degeneration in the myocardial bundles with a 10 mg/kg dose. **e** Congestion in the myocardial blood vessels with 20 mg dose group. **f, g** TCZ-alone groups showed no histopathological changes with both 10 mg/kg and 20 mg/kg doses. **h, I** Ustekinumab (UST)-treated groups showed no histopathological changes with both 10 mg/kg and 20 mg/kg doses

To detect any potential cardiotoxic effect of TCZ, heart tissue specimens were stained and evaluated by H&E examination (Fig. 8). Control samples demonstrated normal histological structures. In contrast, congestion of myocardial blood vessels associated with degeneration in the surrounding myocardium bundles was detected in disease-induced rats. Heart tissue specimens from TCZ-treated rats showed degenerative changes in the myocardial muscle cells with low dose (5 mg/kg), thickening of the myocardial blood vessels, and sclerosis in the vascular wall associated with degeneration in the myocardial bundles with medium dose (10 mg/kg), and congestion in the myocardial blood vessels with high dose (20 mg/kg). Both TCZ-alone and UST (10 mg/kg, 20 mg/kg)-treated groups showed no histopathological changes.

Investigation of cardiac markers such as LDH revealed a significant increase of 206.9% in the disease group compared to the control group. Marked decreases were achieved by both high doses (20 mg/kg) of TCZ and UST groups by 108.7% and 119%, respectively, when compared to the DSS-treated group. The scoring of histopathological alteration is presented in Table 4.

In addition to that, investigation of the Ck-MB marker revealed a significant increase of 186.6% in the disease group compared to the control group. Marked decreases were achieved by both high doses (20 mg/kg) of TCZ and UST groups by 104.8% and 116.8%, respectively, when compared to the DSS-treated group.

**Fig. 9:**
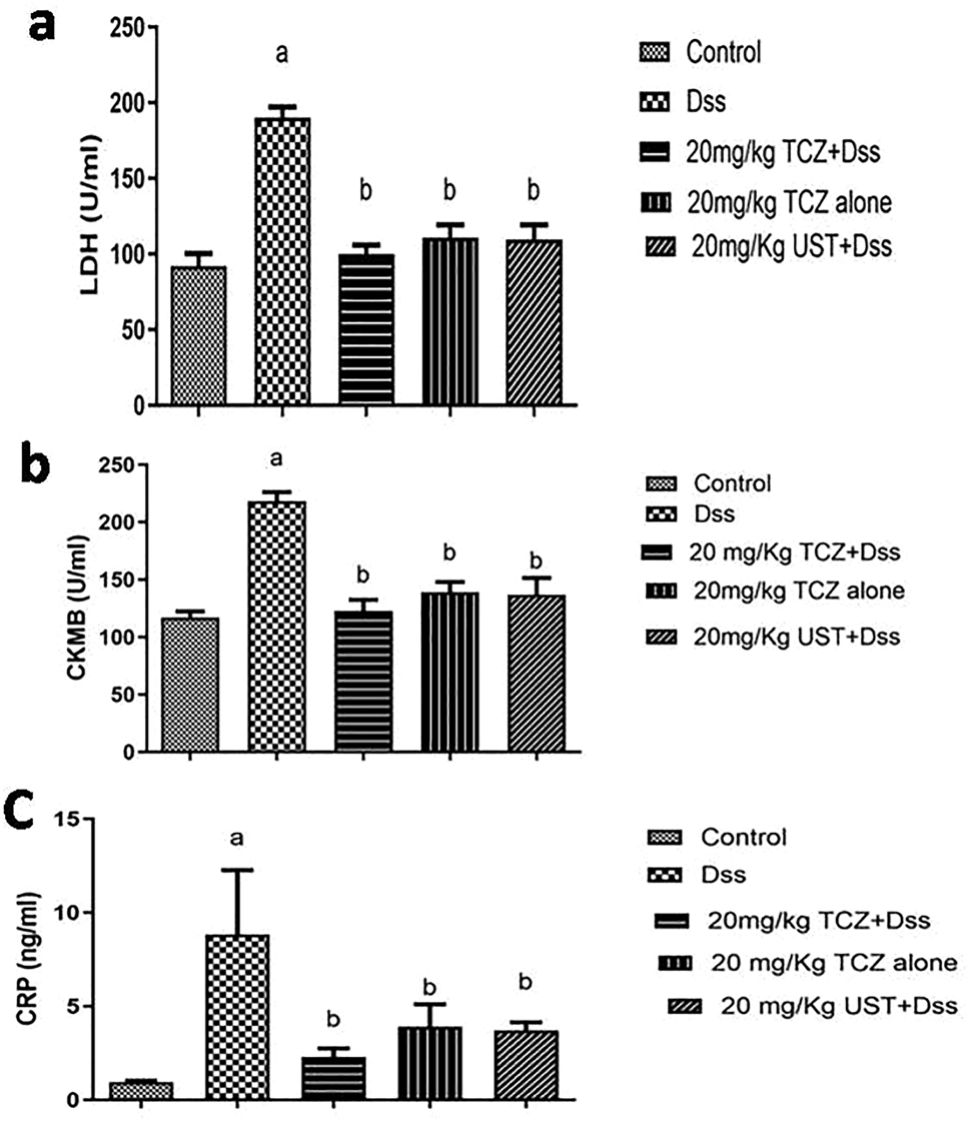
Cardiac markers in serum samples from different treatment groups: dextran sodium sulfate (Dss) group showing higher levels of expression of all three markers (**a** lactate dehydrogenase (LDH), **b** c-reactive protein (CRP), and **c** creatine kinase-myocardial band (CK-MB)) compared to the control group in serum samples while high dose of Tocilizumab (TCZ)-treated groups, TCZ-alone and Ustekinumab (UST)-treated groups showing significant reduction of the expression levels of the three markers compared to Dss group. Data represented with *P* < 0.001 and F (9.785) for CRP and *P* < 0.0001 and *F* (74.41) for LDH and *P* < 0.0001 and *F* = 52.58 for CK-MB (one-way ANOVA followed by Tukey’s multiple comparison post hoc test)

In addition, investigation of CRP marker levels revealed a significant increase of 933.4% in the disease group compared to the control group. The marked decrease was achieved by both high doses (20 mg/kg) of TCZ and UST groups by 242.1% and 389.5%, respectively, when compared to the DSS-treated group. No significant difference in expression levels of all three markers between TCZ- and UST-treated groups at respective doses. All results are presented for three cardiac markers: LDH, CK-MB, and CRP in (Fig. 9A, B, C), respectively.

## Discussion

IBDs are autoimmune diseases and complex inflammatory disorders affecting the gastrointestinal tract including UC and CD. UC is an idiopathic, chronic, relapsing–remitting inflammatory condition, which is accompanied by diarrhea, bloody stools, frequent bowel movements, abdominal pain, fever, malnutrition, and weight loss. It can eventually progress to toxic megacolon, colonic perforations, and colon cancer (Tavakoli et al. 2021). The pathogenesis of UC is complex and involves several factors such as immune, genetic, and environmental (such as diet, breastfeeding, medication use, vitamin D status, cessation of smoking, and bacterial infections) factors (Huang and Chen 2016; Kaur and Goggolidou 2020).

DSS-induced colitis has been used on a wide scale in research (Randhawa et al. 2014) to induce intestinal colitis in mice (Chassaing et al. 2014) and rats (Matuszyk et al. 2015; Senol et al. 2015; Lleal et al. 2019). It is a morphologically and symptomatically resembling model for ulcerative colitis in humans. DSS has a direct toxic effect on the inner mucus layer, leading to intestinal erosions and ulcerations accompanied by bacterial penetration and antigens before any inflammatory signs can be seen. Thus, it can be concluded that a loss of the inner colon mucus layer is the initial episode leading to bacterial penetration and ultimately, the development of an inflammatory response (Gaudio et al. 1999). Accordingly, DSS has been applied in the study of innate immune mechanisms involved in the pathogenesis of IBDs (Low et al. 2013).

Importantly, a huge amount of research has targeted the role of Interleukins and proposed that IL-6 might play an important role in the development of IBD (Lee et al. 2012; Tanaka al. 2014). IL-6 has multiple functions depending on its signaling pathway as it binds with two types of receptors: membrane-bound IL-6 receptor (mIL-6R) and soluble IL-6 receptor (sIL-6R) (Rose-John and Neurath 2004). IL-6 has two different features based on its signaling pathway including pro-inflammatory features that are correlated to the trans-signaling mechanism and anti-inflammatory features that are secondary to the classic signaling mechanism (Kishimoto 2006). Hence, in this study, we investigated the colo-protective and immunomodulatory effect of the monoclonal IL-6 antibody TCZ as a therapeutic agent in a Wistar rat model of DSS-induced UC.

First, in the current study, the administration of DSS resulted in significant ulceration, congestion, and edema as signs of colitis development with subsequent increased ulcerative lesion score, and presence of blood in stool. TCZ doses reduced this effect as decreased diarrhea, increased colon length, and decreased stool consistency. In accordance with the current results, a previous case of a patient with coexisting UC and rheumatoid arthritis confirmed the successful use of TCZ on clinical scores of UC (Szeto et al. 2016). In contrast, there were cases in which the remission was not induced by TCZ which were treated for Takayasu arthritis and relapsing polychondritis (Hanioka et al. 2021). Second, histopathological examination of the colon tissues, in our study, confirmed our aforementioned experimental results. Disease-induced colons were associated with histological abnormalities such as focal necrosis and ulceration with inflammatory cell infiltration in the mucosa and submucosal layers. In addition, hypertrophy with few inflammatory cells’ infiltration in the muscularis, and thickening and edema in the serosal layer. Successfully, treatment with TCZ effectively ameliorated DSS-induced injury. Briefly, colon tissue specimens from TCZ and UST groups showed normal histological structure of the mucosal layer with lamina propria, submucosa, muscularis, and serosa.

Inflammation and inflammatory cell infiltration play a fundamental role in the pathophysiology of UC (Hendrickson et al. 2002). In the current study, DSS-inducing UC significantly increased levels of IL-6 cytokine in comparison with the control group. Treatment with TCZ and UST showed a significant decrease in IL-6 levels (Narazaki and Kishimoto 2018) compared with the DSS group. In agreement with our results (Choy et al. 2020) observed, TCZ bind to the IL-6 receptor and block signaling of the pro-inflammatory cytokine IL-6. Furthermore, STAT-3 plays an important role in the pathophysiology of IBD: in response to infection or injury, it is rapidly activated within cells mainly via cytokines, of the IL-6 family, to facilitate the return to homeostasis. However, STAT-3 causes chronic inflammation with continuous activation (Kasembeli et al. 2018). Analysis of the STAT-3 inflammatory marker, in our study, revealed that DSS-induced colitis in rats showed a significant increase in STAT-3 expression in comparison with the control group. Both TCZ and UST as therapeutic agents showed significant decreases in STAT-3 expression compared with the DSS group. TCZ was reported to prevent joint destruction and improve rheumatoid arthritis symptoms, by down-regulating the expression of the receptor activator of NF-kappa ligand (RANKL) involved in rheumatoid bone destruction, by blocking IL-6 trans-signaling. In the absence of TCZ, RANKL was induced by IL-6/soluble IL-6 receptor complex via the Janus Kinase (JAK)/STAT signaling pathway (Hashizume et al. 2008). Indeed, targeting the IL-6/ STAT-3 signaling pathway may represent a viable treatment strategy for IBD (Mitsuyama et al. 2007).

Apoptosis is one of the programmed cell deaths reported to be a contributor to UC pathogenesis (Nunes et al. 2014; Becker et al. 2013). Apoptosis induced due to certain pro-apoptotic markers, such as Bax, promotes cytochrome c release to activate caspase-3 (O'Brien and Kirby 2008). In the present study, DSS switched on the apoptotic pathway as showed by increasing levels of caspase-3, while TCZ and UST treatments prevented the DSS-triggered apoptosis as shown by the suppressed caspase-3 levels. This finding is consistent with the previous study by Xu et al. (2005) in which “garcilin” showed inhibition of apoptosis of enterocytes and lymphocytes in a rat model of colitis through inhibiting levels of bcl-2 and baxproteins as a mechanism to protect against the damage of UC.

ER stress triggers the activity of ER membrane resident proteins: IRE-1 and ATF-6 that sense the presence of unfolded proteins in the ER lumen (Kaser and Blumberg 2009; Cao 2016). Therefore, to evaluate the effect of ER stress in the development of inflammation in UC-induced rats and the potential effect of TCZ treatment, we assessed ER stress proteins (IRE-1 and ATF-6) in colon tissue specimens of all experimental groups by Western blot technique. Exploring the mechanism of the ER stress in the secretory cells of Intestinal endothelial cells (IECs) may add a missing piece to the puzzle of IBD therapy. The current study showed that DSS significantly increased expression levels of ER stress signaling proteins: IRE-1 and ATF-6 as compared with their respective control group. Treatment with TCZ or UST in the current study significantly attenuated DSS-induced elevation in both proteins' expression levels. This finding is consistent with a previous study, where glutamine was used as a treatment in Trinitrobenzene sulfonic acid (TNBS)-induced colitis in rats and was able to attenuate ER stress signaling and protect against damage caused by UC (Crespo et al. 2012).

Autophagy plays multiple roles in IBD pathogenesis (Iida et al. 2017). It alters processes including bacterial killing, pro-inflammatory cytokine production by macrophages, antigen presentation by dendritic cells, and the ER stress response (Hosomi et al. 2015). The current study examined ATG16L1 and NOD2 expressions, which significantly decreased ATG16L1 and NOD2 expression levels in the DSS-induced colitis rats compared with their respective control groups. In contrast, TCZ and UST treatments significantly increased ATG16L1 and NOD2 expressions. Following our study, curcumin, by targeting and suppressing the autophagy pathway, ameliorated UC symptoms in DSS-induced colitis in mice (Yue et al. 2019). In addition, Berberine, as a clinical anti-diarrhea and anti-inflammatory drug, showed that it can significantly inhibit the expression and secretion of lysozyme by promoting autophagy via the AMPK/MTOR/ULK1 pathway in DSS-induced UC mice model (Xu et al. 2022).

To investigate the potential toxicity of TCZ on the cardiovascular system, in the present study, heart tissue specimens were taken and stained by H&E. Congestion in the myocardial blood vessels associated with degeneration in the surrounding myocardium bundles was observed in DSS-induced colitis rats. While TCZ-treated rats showed degenerative changes in the myocardial muscle cells with the low dose (5 mg/kg), thickening of the myocardial blood vessels, and sclerosis in the vascular wall associated with degeneration in the myocardial bundles with the medium dose (10 mg/kg), and congestion in the myocardial blood vessels with the high-dose (20 mg/kg) group. However, heart tissue specimens from rats that administered only TCZ and also administered UST showed no histopathological alteration with both medium and high doses. The safety of TCZ was confirmed in the present work by attenuating the DSS-mediated elevation of the cardiac markers: LDH, Ck-MB, and CRP. In addition, these markers showed a decrease with UST high dose.

## Conclusion

**Fig. 10:**
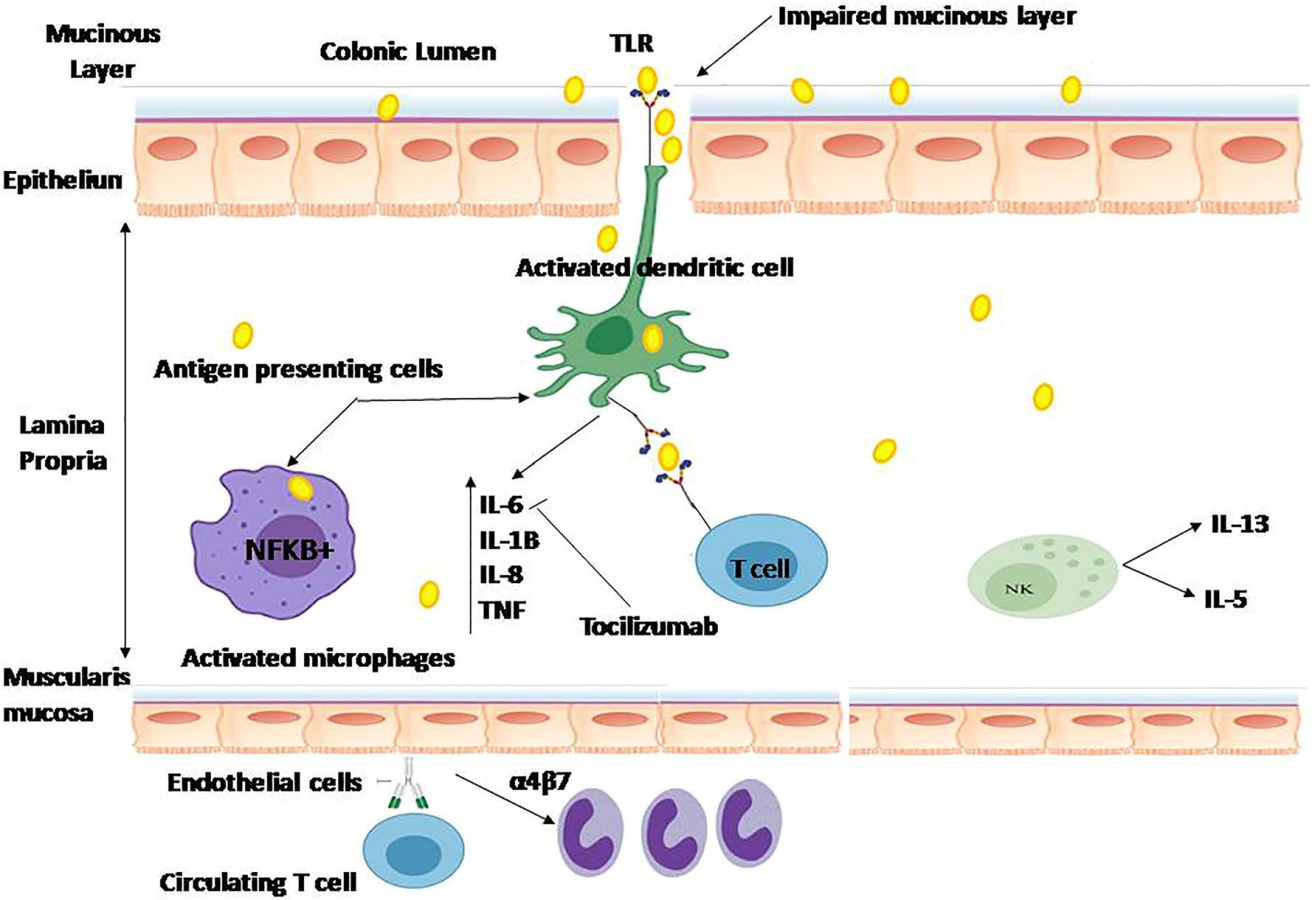
Signaling events in ulcerative colitis: ulcerative colitis (UC) signaling development starts when antigens bind to and activate toll-like receptors (TLRs) to trigger immune cells such as dendritic cells and macrophages and activate the signaling pathway of nuclear factor kappa (NFkβ), with subsequent production of several types of cytokines. Antigen interacts with T cells causing activation and differentiation into Th-17 which produces interleukin-17 to promote inflammation. Other cytokines such as interleukin-6 (IL-6) induce immune cell differentiation (B-Cells) resulting in the production of autoantibodies. Furthermore, IL-6 and tumor factor necrosis-alpha (TNF-α) together with other cytokines enhance inflammation through the production of vascular cell adhesion molecule-1 (VCAM-1), very late antigen-4 (VLA4) and intracellular adhesion molecule-1 (ICAM-1). In addition, circulating T cells have α4β7 which binds to endothelium expressing mucosal vascular address cell adhesion molecule (MADCAM) resulting in increased inflammation as a consequence of increased entering of gut-specific T cells. Tocilizumab (TCZ) blocks signal transduction of IL-6 for the treatment of UC

Our study highlights the promising colo-protective and immunomodulatory effect of TCZ, hence reducing inflammation and other symptoms of UC. Interestingly, TCZ effectively ameliorated DSS-induced injury via decreasing inflammatory markers of colon injury (IL-6, STAT-3, and CRP) and apoptosis pathway via Caspase-3 and ER stress sensor proteins; inositol-requiring transmembrane kinase endonuclease-1 (IRE-1) and activated transcription factor-6 (ATF-6) and autophagy proteins; ATG16L1 and nucleotide-binding oligomerization domain-containing protein-2 (NOD2) as depicted in Fig. 10. Furthermore, a favorable safety profile on the cardiovascular system was demonstrated by the improved histopathology of cardiac tissue especially with high doses of TCZ in addition to the reduced expression levels of cardiac markers (LDH, Ck-MB and CRP). Hence, the current findings mark the colo-protective effect of TCZ as a promising therapy for UC.

## Limitations and future directions

1. This study was conducted on one strain of rats, hence re-evaluating the effect of TCZ on other strains of rats and mice and other experimental animals may be considered as a future direction for better assessment of the colo-protective effect of TCZ.
2. Assessment of TCZ in a clinical study of UC patients would be a valuable addition to provide a more comprehensive picture of the reported effect in this manuscript.

## Data Availability

Data are avaialable upon reasonable request.

## Abbreviations

**ADA**: Anti-drug antibody
**AMPK**: Adenosine monophosphate-activated protein kinase
**ANOVA**: Analysis of variance
**ASU**: Ain Shams University
**ATF-6**: Activated transcription factor-6
**ATG16L1**: Autophagy-related 16-like 1 protein
**CD**: Crohn’s disease
**CK-MB**: Creatine kinase-myocardial band
**CRP**: C-reactive protein
**DAI**: Disease activity index
**DMARDs**: Disease-modifying anti-rheumatic drugs
**Dss**: Dextran sodium sulfate
**ELISA**: Enzyme-linked immunosorbent assay
**ER**: Endoplasmic reticulum
**H & E**: Hematoxylin and eosin
**HF**: Heart failure
**HRP**: Horseradish peroxidase
**IBD**: Inflammatory bowel diseases
**ICAM-1**: Intracellular adhesion molecule-1
**IECs**: Intestinal endothelial cells
**IL-1β, IL-6, IL-8, IL-12, IL-23**: Interleukins
**I.p**: Intraperitoneal
**IRE-1**: Inositol requiring transmembrane kinase endonuclease-1
**JAK**: Janus kinase-1
**Kg**: Kilogram
**LDH**: Lactate dehydrogenase
**Mg**: Milligram
**mIL-6R**: Membrane-bound IL-6 receptor
**mTOR**: Mammalian target of rapamycin
**NFK**: Nuclear factor kappa
**Ng**: Nano-gram
**NOD2**: Nucleotide-binding oligomerization domain-containing protein-2
**NODCAR**: National Organization for Drug Control and Research
**Pg**: Picogram
**RANKL**: Receptor activator of nuclear factor kappa beta ligand
**SD**: Standard of deviation
**SDS-PAGE**: Sodium dodecyl sulfate–polyacrylamide gel electrophoresis
**sIL-6R**: Soluble IL-6 receptor
**STAT-3**: Signal transducer and activator of transcription-3
**TCZ**: Tocilizumab
**TLRs**: Toll-like receptors
**TNBS**: Trinitrobenzene sulfonic acid
**TNF-α**: Tumor necrosis factor-α
**UC**: Ulcerative colitis
**UST**: Ustekinumab
**VCAM-1**: Vascular cell adhesion molecule-1
